# Supplementary material for: Coexistence of multiple metastable polytypes in rhombohedral bismuth
Source: Sci Rep. 2016 Feb 17;6:20337. doi: 10.1038/srep20337 (PMC4756663; doi:10.1038/srep20337)
Supplement: Supplementary Information [file srep20337-s1.pdf]

Coexistence of multiple metastable polytypes in rhombohedral bismuth

Yu Shu[1], *Wentao Hu*[1], *Zhongyuan Liu*[1], *Guoyin Shen*[2], *Bo Xu*[1], *Zhisheng Zhao*[1], *Julong He*[1], *Yanbin Wang*[3], *Yongjun Tian*[1], *Dongli Yu*[1]*

[1]State Key Laboratory of Metastable Materials Science and Technology, Yanshan University, Qinhuangdao, Hebei 066004, China

[2]High Pressure Collaborative Access Team, Geophysical Laboratory, Carnegie Institution of Washington, Argonne, Illinois 60439, USA

[3]Center for Advanced Radiation Sources, University of Chicago, Argonne, Illinois 60439, USA

Supplemental Materials

Table S1. Details of the HP-HT treatment conditions.

Sample no.	Pressure (GPa)	Temperature K	Sample state under treatment conditions
1	2±0.1	300±5	Phase - I
2	2.7±0.1	300±5	Phase - II
3	2±0.1	1073±10	Liquid
4	2±0.1	2273±30	Liquid

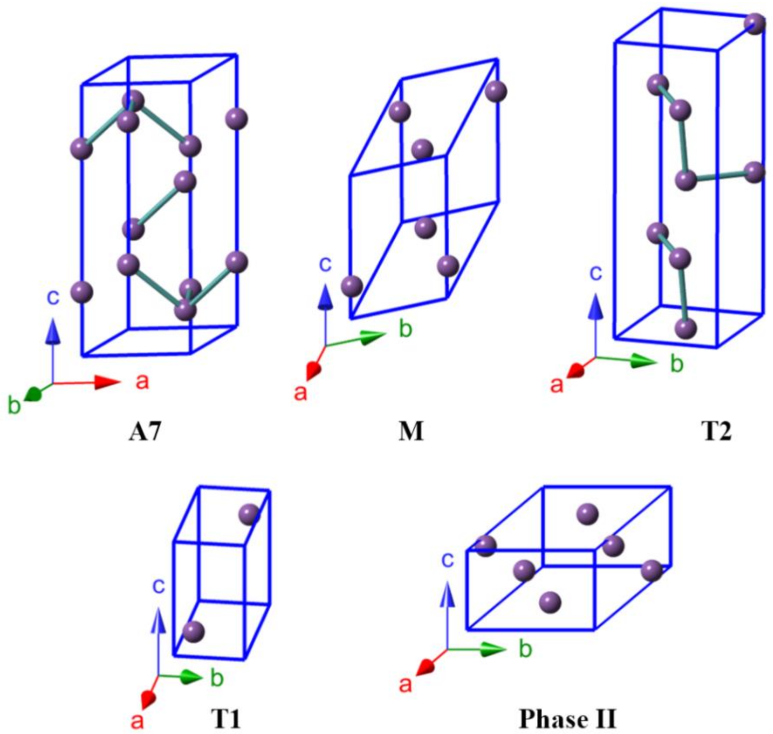

FIG. S1. Unit cells of the Bi crystal structures.

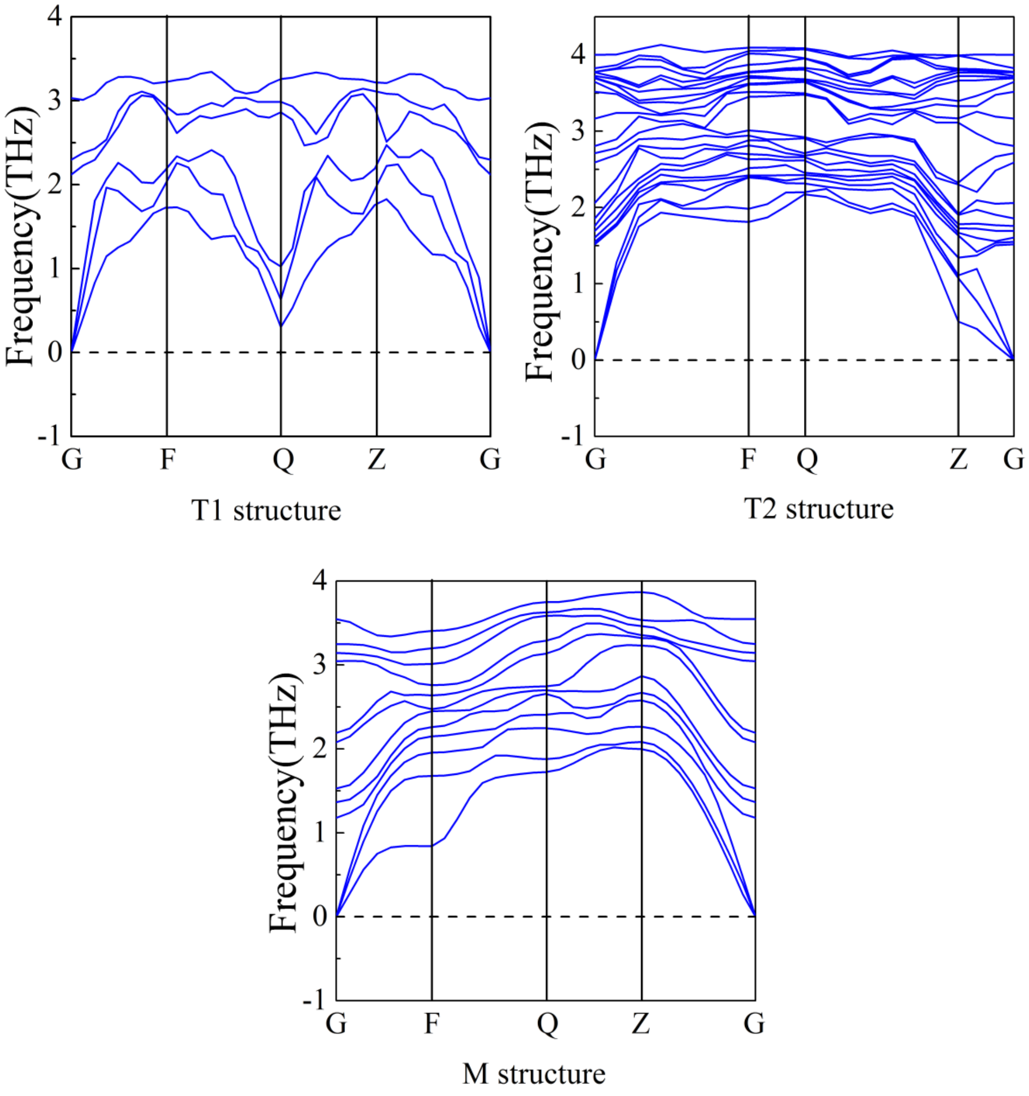

FIG. S2. Phonon spectra of new Bi polytype structures.
